# Factor Xa Inhibition with Apixaban Does Not Influence Cardiac Remodelling in Rats with Heart Failure After Myocardial Infarction

**DOI:** 10.1007/s10557-020-06999-7

**Published:** 2020-05-26

**Authors:** Salva R. Yurista, Herman H. W. Silljé, Kirsten T. Nijholt, Martin M. Dokter, Dirk J. van Veldhuisen, Rudolf A. de Boer, B. Daan Westenbrink

**Affiliations:** Department of Cardiology, University Medical Center Groningen, University of Groningen, PO Box 30.001, Groningen, 9700 RB The Netherlands

**Keywords:** Anticoagulant, Heart failure, Cardiac function, Cardiac remodelling

## Abstract

**Background:**

Heart failure (HF) is considered to be a prothrombotic condition and it has been suggested that coagulation factors contribute to maladaptive cardiac remodelling via activation of the protease-activated receptor 1 (PAR1). We tested the hypothesis that anticoagulation with the factor Xa (FXa) inhibitor apixaban would ameliorate cardiac remodelling in rats with HF after myocardial infarction (MI).

**Methods and Results:**

Male Sprague-Dawley rats were either subjected to permanent ligation of the left ascending coronary artery (MI) or sham surgery. The MI and sham animals were randomly allocated to treatment with placebo or apixaban in the chow (150 mg/kg/day), starting 2 weeks after surgery. Cardiac function was assessed using echocardiography and histological and molecular markers of cardiac hypertrophy were assessed in the left ventricle (LV). Apixaban resulted in a fivefold increase in anti-FXa activity compared with vehicle, but no overt bleeding was observed and haematocrit levels remained similar in apixaban- and vehicle-treated groups. After 10 weeks of treatment, LV ejection fraction was 42 ± 3% in the MI group treated with apixaban and 37 ± 2 in the vehicle-treated MI group (*p* > 0.05). Both vehicle- and apixaban-treated MI groups also displayed similar degrees of LV dilatation, LV hypertrophy and interstitial fibrosis. Histological and molecular markers for pathological remodelling were also comparable between groups, as was the activity of signalling pathways downstream of the PAR1 receptor.

**Conclusion:**

FXa inhibition with apixaban does not influence pathological cardiac remodelling after MI. These data do not support the use of FXa inhibitor in HF patients with the aim to amend the severity of HF.

**Figure:**
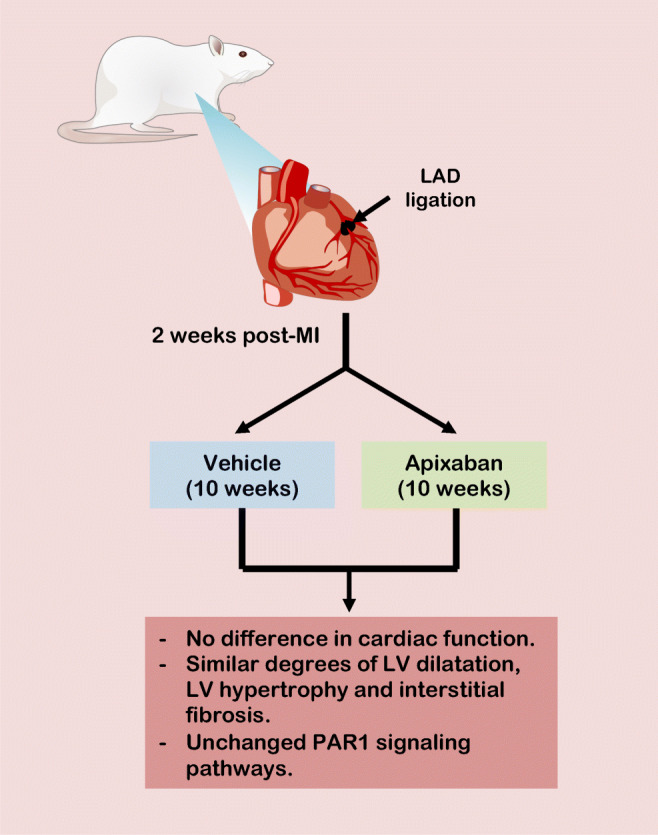
Graphical Abstract

**Electronic supplementary material:**

The online version of this article (10.1007/s10557-020-06999-7) contains supplementary material, which is available to authorized users.

## Introduction

Heart failure is a major global health problem that is reaching epidemic proportions in the near future [1, 2]. Despite the large range of pharmacological and device-based therapies available, mortality and morbidity remain high [3]. Patients with heart failure (HF) are at increased risk of stroke and other thromboembolic events and are also more likely to succumb from these events [4–9]. The higher incidence of thromboembolic events is observed both in patients with sinus rhythm with atrial fibrillation [10, 11], suggesting that HF should be considered as a prothrombotic or hypercoagulable state. Interestingly, coagulation factors such as thrombin and FXa can exert direct effects on the heart, which are thought to promote inflammation, endothelial dysfunction and maladaptive cardiac remodelling [12]. Anticoagulants could therefore offer therapeutic benefits in HF patients beyond the prevention of thromboembolic events [10].

The effects of thrombin and FXa on myocardial tissues are thought to be governed by protease-activated receptors (PARs), which coordinate a myriad of cellular responses in multiple cell types. PAR1 and PAR2 are expressed in cardiac tissue and it has been proposed that these receptors contribute to the progression of HF [13–15]. Indeed, cardiomyocyte-specific overexpression of PAR1 induces cardiac hypertrophy that rapidly progressed into dilated cardiomyopathy [16]. Conversely, deletion of the PAR1 receptor attenuates cardiac remodelling after a myocardial infarction (MI) in mice [16]. Nevertheless, direct evidence for enhanced activity of PARs in HF is sparse and the exact role of PAR signalling in cardiac remodelling remains poorly defined. Furthermore, it is unknown whether PAR receptor activation in HF is amendable by anticoagulant therapy. In this study, we tested the hypothesis that inhibition of PAR signalling by the direct FXa inhibitor apixaban could attenuate cardiac remodelling in rats with established LV dysfunction after MI.

## Methods

### Experimental Protocol

**Scheme 1:**
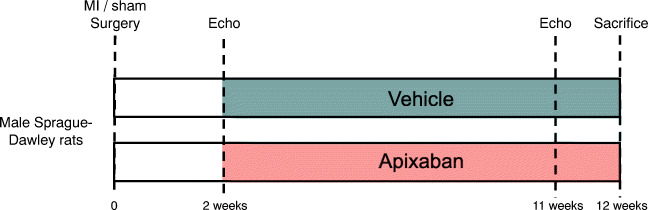
Schematic representation of the experimental protocol

Male Sprague-Dawley rats (Envigo, The Netherlands) were randomized to treatment with chow containing apixaban or control chow, starting 2 weeks after MI surgery. We chose this protocol to represent a population of stable chronic HF after a large myocardial infarction, which still represents the majority of patients with chronic HF. Here, we examined the effects of clinically relevant doses of apixaban on cardiac remodelling in rats with HF after MI [17]. Treatment allocation was stratified according to left ventricular ejection fraction (LVEF) to ensure that the baseline cardiac function is similar in the apixaban and the vehicle groups. After 10 weeks of treatment, rats were anaesthetized, blood was drawn and the hearts were rapidly excised for further analysis. Rats with an infarct size of less than 15% were excluded from analysis as these small infarcts are haemodynamically fully compensated [18]. The experimental protocol is illustrated in Scheme 1.

### Ethical Statement

The experimental protocol was approved by the Animal Ethical Committee of the University of Groningen (IvD number: 16487-02-001). The investigation conforms to the Guide for the Care and Use of Laboratory Animals published by the US National Institutes of Health (NIH publication no. 85–23, revised 1996). We followed ARRIVE guidelines when reporting this study.

### MI Surgery

Rats were randomized to HF or sham surgery under isoflurane (2.5%) inhalation anaesthesia. After left-sided thoracotomy, HF was induced by permanent ligating of the proximal portion of the left coronary artery as previously described [19]. Sham-operated rats underwent the same procedure but without coronary ligation.

### Investigational Drug

Apixaban was kindly supplied by Bristol-Myers Squibb (BMS), USA. Apixaban was mixed with standard rat chow (R/M-H V1534-70, Ssniff, Germany) in a final concentration of 1.95 g/kg intended to reach an average dose of 150 mg/kg. Standard rat chow (R/M-H V1534-70, Ssniff, Germany) was used as the control (vehicle).

### Echocardiography

Two weeks after surgery and 1 week before termination, the M-mode and 2D echocardiography were performed using a Vivid 7 echo machine (GE Healthcare) equipped with a 10-MHz phase array linear transducer for serial assessment of cardiac structure and function as previously described [19].

### Invasive Haemodyamic Measurements

Prior to sacrifice, invasive haemodynamics were analysed by aortic and LV catheterization as previously described [19]. The right carotid artery was isolated and punctured and a 1.9-F rat pressure-volume catheter (Scisense, London, Ontario, Canada) was inserted into the right carotid artery. The tip of the catheter was advanced through the aorta into the LV cavity. Heart rate (HR), left ventricular end-systolic (LVESP) and end-diastolic (LVEDP) pressures, and maximal rates of increase and decrease in developed LV pressure (dP/dtmax and dP/dtmin) were determined. The data were acquired using a PowerLab data acquisition system (ADInstruments, Colorado Springs, CO) and analysed with a LabChart 8 software.

### Infarct Size, Cardiomyocyte Size and Interstitial Fibrosis Measurement

Rats were euthanized under isoflurane anaesthesia. The heart was rapidly excised and weighed. The mid-papillary slice of the LV was fixed in 4% formaldehyde and paraffin-embedded. The infarct size was calculated using midline length methods as the percentage of the scar length to the total LV circumference on Masson’s trichrome–stained section, as described previously [19, 20]. Furthermore, Masson’s trichrome staining was also used to evaluate the extent of interstitial fibrosis. A Hamamatsu microscope was used to capture the whole tissue section and an Aperio ImageScope software was used to quantify fibrosis in the non-infarcted LV [19]. Finally, sections were stained with FITC-labelled wheat germ agglutinin (WGA) to determine cardiomyocyte size as previously described [19]. Cell size from transversally cut cardiomyocytes in the non-infarcted LV was measured using an image analyser (Zeiss KS400, Germany) and a quantified using ImageJ software (National Institutes of Health, Bethesda, MD, USA). The investigators analysing the data were blinded to the treatment allocation.

### Blood and Urine Measurements

Blood samples were obtained via a tail vein under isoflurane anaesthesia to determine anti-factor Xa activity (AXA), prothrombin time (PT) and activated partial thromboplastin time (APTT) levels. At sacrifice, 8 ml of blood was drawn from the abdominal aorta (either anti-coagulated with sodium citrate or EDTA), and urine was collected directly from the bladder. Complete blood count was determined on the day of sacrifice using a Sysmex Hematology Analyzer (Symex XN-10, Sysmex Corporation, Japan).

### Prothrombin Time, Activated Partial Thromboplastin Time and Anti-Factor Xa Activity Assay

To determine the effect of apixaban on PT and APTT levels, each sample was tested using Innovin (PT) and Actin FS (APTT) reagents (Siemens Healthcare Diagnostics). Plasma apixaban concentration was assessed using a chromogenic anti-factor Xa activity (AXA) assay, the Berichrom Heparin Assay (Dade Behring, Marburg, Germany) as this is the most reliable method to measure the pharmacodynamics of apixaban [21].

### Urine Occult Blood Test

Haemoglobin/red blood cells were determined by a semiquantitative method using Combur10 Test Sticks (Roche Diagnostics GmbH, Mannheim, Germany).

### Quantitative Real-time PCR

RNA was extracted from the non-infarcted LV using TRIzol reagent (Invitrogen Corp., Carlsbad, CA, USA), as previously described [19, 22] and the NanoDrop device was used to measure RNA concentration. Random primer mix was used to prepare first-stranded DNA and thereafter used as a template for quantitative real-time reverse-transcriptase PCR (qRT-PCR) (25 ng/reaction). mRNA levels obtained by a qRT-PCR using a C1000 Thermal Cycler CFX384 Real-Time PCR Detection System (Bio-Rad Laboratories, Veenendaal, The Netherlands). 36B4 reference gene was used to correct all measured mRNA expression. Primer sequences can be found in Supplementary table S1.

### Western Blot

Frozen non-infarcted LV tissue was homogenized in ice-cold lysis buffer containing phosphatase inhibitor cocktail 1 (Sigma) and protease inhibitor (ROCHE) as described previously [19]. Bio-Rad DC Protein Assay (Bio-Rad Laboratories, Veenendaal, The Netherlands) was used for protein quantifications with bovine albumin as a standard, as described before [19]. Immunoblotting was performed using primary antibodies from commercial suppliers (Supplementary table S2). Immunoblots were incubated with appropriate secondary antibodies for 1 h at room temperature. Signals were detected by ECL (ParkinElmer, Waltham, MA, USA). Blots were quantified using ImageJ software (National Institutes of Health, Bethesda, MD, USA). The density of each band was normalized to GADPH acting as a loading control and presented as fold change over Sham-veh group.

### RhoA Activity Assay

RhoA activity was measured according to the manufacturer’s protocol (BK124; Cytoskeleton Inc.), as previously described [23].

### Statistical Analysis

Data are presented as means ± standard errors of the mean (SEM). To compare normally distributed parameters, one-way analysis of variance (ANOVA) followed by Tukey’s post hoc test was used. When data were not normally distributed, a non-parametric Kruskal-Wallis test followed by a Mann-Whitney *U* test with correction for multiple comparisons was used. Wilcoxon signed-rank test was used to evaluate LVEF post-MI vs before termination. Differences were considered significant at *p* < 0.05. IBM SPSS Statistics for Windows, Version 23.0 (IBM Corp, USA), was used to perform all statistical analysis.

## Results

We performed MI or sham surgery on a total of 78 male Sprague-Dawley rats; 21 rats (27%) died during the surgical procedure; all remaining rats survived the rest of the study. A total of 4 rats with an infarct < 15% of the LV were excluded from further analysis, leaving a total of 53 rats for the analysis. The final group sizes were 8 for the sham-apixaban group, 13 for the sham-vehicle group, 17 for the MI-apixaban group and 15 for the MI-vehicle group.

### Efficacy and Safety of Apixaban in Rats with HF After MI

#### Efficacy of Apixaban

Daily food intake and water intake were comparable among the groups (Table 1). As expected, plasma PT and APTT were not affected by apixaban treatment (Table 1) [24]. In rats treated with apixaban, the plasma AXA activity was increased by fivefold as compared with that in vehicle-treated rats (Table 1).

**Table 1:** General characteristics and haematological parameters

Parameters	Sham-vehicle	Sham-apixaban	MI-vehicle	MI-apixaban
Food intake (g/day)	31.9 ± 0.5	29.9 ± 0.7	30.3 ± 0.5	29.6 ± 0.3
Water intake (ml/day)	32.0 ± 0.9	29.9 ± 0.6	30.9 ± 1.0	28.5 ± 0.4
Body weight change (g)	91.15 ± 3.2	93.25 ± 4.8	94.73 ± 4.8	93.24 ± 4.8
AXA (ng/ml)	21 ± 2	109 ± 17^†^	19 ± 1	103 ± 11^#^
PT (s)	11.12 ± 0.12	11.00 ± 0.10	11.31 ± 0.21	11.17 ± 0.17
APTT (s)	15.48 ± 1.13	16.74 ± 0.62	17.24 ± 1.20	16.91 ± 0.94
WBC (10^9/l)	9.34 ± 2.18	11.34 ± 0.68	11.23 ± 1.24	10.66 ± 0.80
RBC (10^12/l)	9.01 ± 0.17	8.94 ± 0.10	8.99 ± 0.10	9.32 ± 0.23
HCT (mmol/l)	10.02 ± 0.25	9.84 ± 0.12	9.79 ± 0.13	10.00 ± 0.12
HGB (l/l)	0.503 ± 0.02	0.489 ± 0.01	0.493 ± 0.01	0.505 ± 0.01
PLT (10^9/l)	1025.60 ± 53.50	911.00 ± 47.85	826.56 ± 57.10	946.50 ± 57.56

Data are presented as means ± SEM

^†^*p* < 0.05 vs Sham-veh

^#^*p* < 0.05 vs MI-veh

*AXA*, anti-factor Xa activity; *PT*, prothrombin time; *APTT*, activated partial thromboplastin time; *WBC*, white blood cell count; *RBC*, red blood cell count; *HCT*, haematocrit; *HGB*: haemoglobin; *PLT*, absolute automated platelet count

#### Safety of Apixaban

We did not observe any bleeding events during the study, nor did we observe reductions in haemoglobin levels (Table 1) or detect blood in the urine (Supplementary fig. S1). Furthermore, apixaban did not affect other haematological parameters such as white blood cells (WBC), red blood cells (RBC), haematocrit (HCT) and platelet (PLT) counts (Table 1). Body weight was similar in apixaban- and vehicle-treated groups (Table 1).

### Effect of Apixaban on Cardiac Function

**Fig. 1:**
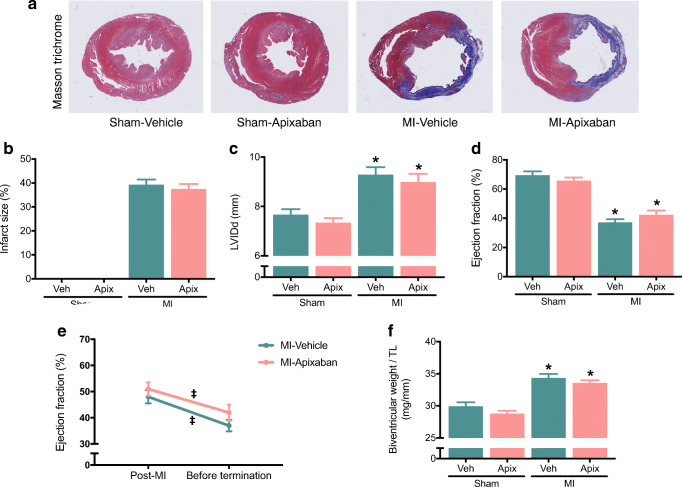
Effect of apixaban on cardiac function. **a** Representative LV sections stained with Masson’s trichrome. **b** Quantification of infarct size from Masson’s trichrome–stained section; *n* = 8–17/group. **c** Left ventricular internal dimensions in diastole (LVIDd) at week 11; *n* = 8–17/group. **d** Ejection fraction of the LV at week 11. **e** Longitudinal change of LV ejection fraction post-MI and before termination; *n* = 8–17/group. **f** Ratio of biventricular weight to tibia length; *n* = 8–17/group. Data are presented as means ± SEM. *p < 0.05 vs sham with the same treatment; ^‡^*p* < 0.05 vs MI—post-MI

The average MI size was 38 ± 2% and was comparable between MI-vehicle and MI-apixaban (Fig. 1a, b). MI surgery resulted in cardiac dilatation and a marked reduction in LVEF (Fig. 1b, c). At the initiation of therapy, 2 weeks after MI, all indices of left ventricular function were comparable between the MI-Apixaban and the MI-vehicle groups. As expected, a progressive deterioration in LVEF was observed in the MI-vehicle group over the 10-week treatment period (Fig. 1e). Treatment with apixaban did not alter cardiac function and all echocardiographic parameters remained comparable between the MI-vehicle- and the MI-apixaban-treated animals (Fig. 1, Table 2). All other relevant echocardiographic parameters at week 11 are depicted in Table 2. The infarcted rats were haemodynamically compromised, as reflected by a decrease in contractility and relaxation (dP/dt max-min), and an increase in LVEDP. Treatment with apixaban did not alter these parameters (Table 3). Moreover, no significant difference was found in systolic blood pressure or diastolic blood pressure among the groups (Table 3).

**Table 2:** Echocardiography parameters in sham-operated and post-myocardial infarction rats at week 11

	Sham-veh	Sham-apixaban	MI-vehicle	MI-apixaban
IVSd (mm)	2.09 ± 0.15	2.46 ± 0.13	1.89 ± 0.10*	1.81 ± 0.13*
LVIDd (mm)	7.67 ± 0.22	7.34 ± 0.18	9.29 ± 0.31*	8.99 ± 0.33*
LVPWd (mm)	2.18 ± 0.13	2.23 ± 0.27	1.88 ± 0.12	1.85 ± 0.12
IVSs (mm)	3.45 ± 0.18	3.64 ± 0.27	2.39 ± 0.15*	2.31 ± 0.21*
LVIDs (mm)	4.52 ± 0.25	4.38 ± 0.22	7.52 ± 0.37*	7.04 ± 0.35*
LVPWs (mm)	3.14 ± 0.13	2.86 ± 0.34	2.37 ± 0.10*	2.42 ± 0.14
FS (%)	41.41 ± 2.37	37.40 ± 1.68	19.02 ± 1.22*	22.17 ± 1.76*
EF (%)	70 ± 2	66 ± 2	37 ± 2*	42 ± 3*

Data are presented as means ± SEM

*p < 0.05 vs sham with the same treatment

*IVS*, interventricular in diastole (d) and systole (s), respectively; *LVID*, left ventricular internal dimensions in both diastole (d) and systole (s); *LVPW*, the thickness of left ventricle posterior wall in diastole (d) and systole (s); *FS*, fractional shortening; *EF*, left ventricular ejection fraction

**Table 3:** Haemodynamic parameters in sham-operated and post-myocardial infarction rats

	Sham-veh	Sham-apixaban	MI-vehicle	MI-apixaban
HR (bpm)	287 ± 15	291 ± 13	299 ± 11	291 ± 8
SBP (mmHg)	119.24 ± 4.37	115.87 ± 6.01	115.77 ± 3.25	110.42 ± 1.93
DBP (mmHg)	84.69 ± 3.45	73.63 ± 2.36	82.66 ± 2.19	75.31 ± 2.46
LVESP (mmHg)	104.49 ± 8.30	110.77 ± 5.78	110.35 ± 3.17	105.94 ± 3.45
LVEDP (mmHg)	12.10 ± 2.24	11.40 ± 1.02	16.30 ± 0.80*	15.85 ± 0.76*
dP/dt max (mmHg/s)	6848 ± 239	6432 ± 504	5427 ± 173*	5021 ± 297*
dP/dt min (mmHg/s)	− 7683 ± 302	− 6766 ± 456	− 5285 ± 224*	− 5304 ± 250*

Data are presented as means ± SEM

*p < 0.05 vs sham with the same treatment

*HR*, heart rate; *bpm*, beat per minute; *SBP*, systolic blood pressure; *DBP*, diastolic blood pressure; *LVESP*, left ventricular end-systolic pressure; *LVEDP*, left ventricular end-diastolic pressure; *dP/dtmax and dP/dtmin*, the maximal rate of increase and decrease of left ventricular pressure, respectively

### Effect of Apixaban on Cardiac Histology and Molecular Markers for Remodelling and Fibrosis

**Fig. 2:**
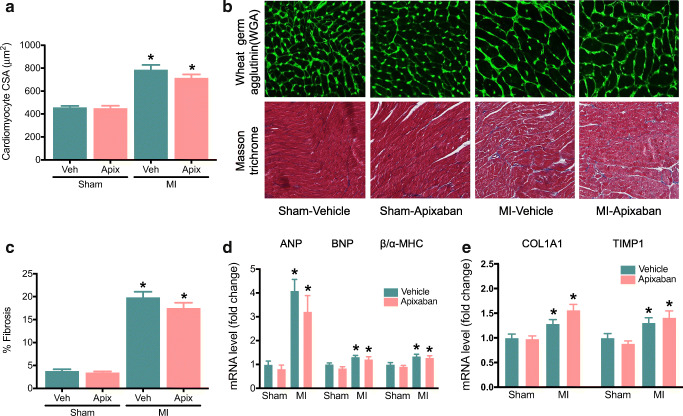
Effect of apixaban on cardiac histology and molecular markers for remodelling and fibrosis. **a** Quantification of cardiomyocyte cross-sectional area from WGA-stained section; *n* = 8–17/group. **b** Representative LV sections stained with WGA and Masson’s trichrome to assess cardiomyocyte hypertrophy and fibrosis. **c** Quantification of fibrosis in non-infarcted LV from Masson’s trichrome–stained section; *n* = 8–17/group. **d**, **e** Measurement of mRNA levels to assess molecular markers for remodelling and fibrosis in non-infarcted LV, respectively, normalized to 36b4; *n* = 8–17/group. Data are presented as means ± SEM. *p < 0.05 vs sham with the same treatment

The biventricular weight/tibia lengths ratio was calculated as a marker of hypertrophy and was found to be significantly increased in MI-vehicle rats compared with that in sham-operated rats (Fig. 1e). The MI rats demonstrated increased cardiomyocyte cross-sectional area and fibrosis compared with the control rats (Fig. 2a–c). However, apixaban therapy did not affect the extent of cardiac hypertrophy nor did it influence the degree of LV fibrosis (Fig. 2a–c).

MI surgery increased the myocardial expression of atrial natriuretic peptide (ANP), and brain natriuretic peptide (BNP) and increased the relative expression of foetal (β-MHC) compared to with that of adult (α-MHC) myosin heavy chain isoform (i.e. β-MHC/α-MHC ratio), as markers for foetal gene reprogramming in heart failure (Fig. 2d). The mRNA levels of cardiac fibrosis markers collagen, type I, alpha 1 (COL1A1) and tissue inhibitor of metalloproteinases 1 (TIMP1) were also significantly increased in the hearts of rats following MI, compared to with that in the hearts of sham rats (Fig. 2e). Apixaban treatment had no effect on ANP, BNP, β-MHC/α-MHC ratio, COL1A1 or TIMP1 mRNA levels (Fig. 2d, e).

### Effect of Apixaban on PAR1 Signalling Pathways

Next, we aimed to determine whether apixaban influenced the activity of thrombin-related pathways downstream of the PAR1 receptor. Binding of thrombin to the PAR1 receptor results in the activation of RhoA, which in turn phosphorylates Rho-associated coiled-coil kinase (ROCK) [25]. The RhoA/ROCK pathway has been shown to contribute to cardiac remodelling in HF [26, 27] It has also been reported that PAR1 activates AKT and ERK, two well-established regulators of cardiac remodelling, independent from RhoA [16, 28].

**Fig. 3:**
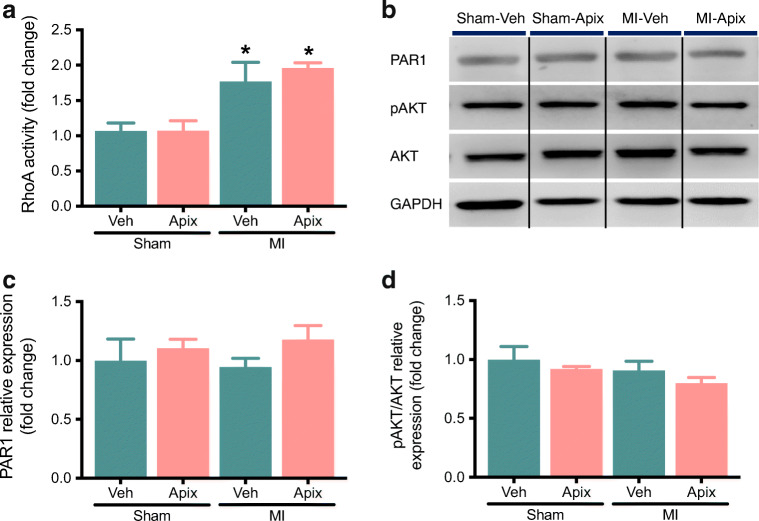
Effect of apixaban on PAR1 signalling pathways. **a** RhoA activity; *n* = 6/group. **b** Western blot analysis of PAR1, total and phosphorylated Akt; *n* = 6/group. **c** Quantification of PAR1 protein levels; *n* = 6/group. **d** Quantification of Akt phosphorylation protein levels; *n* = 6/group. Apix, apixaban. The density of each band was normalized to GADPH acting as a loading control and presented as fold change over Sham-veh group. Data are presented as means ± SEM. *p < 0.05 vs sham with the same treatment

Next, we determined the effects of Apixaban on myocardial RhoA activity. As expected, RhoA activity was increased after MI, but the RhoA activity did not differ between the MI-vehicle and the MI-apixaban group (Fig. 3a). Furthermore, apixaban did not influence the phosphorylation levels of AKT (Fig. 3b, d), nor did it affect the activation of ERK1/2 (data not shown). Finally, the LV protein expression levels of the PAR1 receptor were comparable between all groups (Fig. 3b, c), indicating that the results above could not be explained by aberrant expression of the PAR1 receptor.

## Discussion

We tested the hypothesis that treatment with FXa inhibitors apixaban would improve cardiac function and ameliorate cardiac remodelling in rats with HF after a large transmural anterior MI. For this purpose, we used a well-established model of chronic post-MI HF and ensured that the degree of LV dysfunction was similar in the apixaban and vehicle-treated groups at the initiation of therapy. Furthermore, we used a clinically relevant dose of apixaban that also appeared to be safe and effective, as evidenced by a consistent 5-fold increase in AXA and by the absence of (occult) bleeding. We demonstrate that treatment with apixaban did not influence LV function, nor did it influence LV dilatation of LV hypertrophy. Moreover, histological and molecular markers for pathological LV remodelling were also not influenced by apixaban and the activity of signal transduction pathways downstream of the PAR1 was unaltered. These findings suggest that the inhibition of FXa with a safe, effective and clinically relevant dose of apixaban does not influence cardiac remodelling in the chronic phase after MI. In addition, our findings suggest that the role of thrombin-mediated PAR1 receptor activation to the pathophysiology of HF is limited. Our findings are in line with the results from the COMMANDER HF trial and do not support the use of FXa inhibitor in HF patients with the aim to amend the severity of HF.

HF reflects a pro-coagulant state, because all prerequisites for thrombosis as described in Virchow’s law are met: abnormalities in blood flow, in the vessel wall and in blood constituents [29, 30]. Aberrant platelet activation and increased levels of pro-coagulant factors reflect abnormalities in blood constituents. Impaired contractility and dilated chambers perturb myocardial blood flow and endothelial damage and reduced endothelium-dependent vasodilation reflect the changes in the vessel wall [31]. It has been previously recognized that patients with HF have an increased risk of thromboembolic events, both systemic and venous. The prothrombotic molecules, such as fibrinogen and von Willebrand factors, have been found to be elevated in subjects with HF. Moreover, previous studies showed that the thrombotic risk associated with HF appears to increase with the severity of the disease [32, 33]. A large Danish prospective cohort study demonstrated that the risk of stroke and thromboembolic event was increased in HF patients with HF independent of the CHA2DS2-VASc score. Furthermore, in patients with HF and a CHA2DS2-VASc score ≥ 4, the absolute risk of thromboembolic events was even higher in patients without than with concomitant AF [34]. In addition, the prognosis of HF markedly deteriorates after a thromboembolic event occurs, suggesting that interventions to reduce thromboembolic events may improve prognosis in HF patients [35]. Another mechanism by which the hypercoagulable state could contribute to the progression of HF is coronary microvascular embolization leading to MI [36, 37].

Anticoagulants are often prescribed in HF patients without AF that are considered to be at high risk for stroke, such as those with an LV thrombus. There is, however, very little evidence to support the lenient prescription of anticoagulants in patients with HF and sinus rhythm [38]. In the Warfarin versus Aspirin in Reduced Cardiac Ejection Fraction (WARCEF) trial, warfarin did not result in a meaningful reduction in the rates of ischaemic stroke, intracerebral haemorrhage or death from any cause [39]. Furthermore, the effect of FXa inhibitor rivaroxaban on clinical outcomes was recently tested in the COMMANDER HF (“A Study to Assess the Effectiveness and Safety of Rivaroxaban in Reducing the Risk of Death, Myocardial Infarction, or Stroke in Participants with Heart Failure and Coronary Artery Disease Following an Episode of Decompensated Heart Failure”) trial [40]. The authors randomized > 5000 patients with chronic ischaemic HF with reduced ejection fraction but without atrial fibrillation to a low dose of rivaroxaban or placebo. Rivaroxaban was safe and well tolerated, but did not affect the incidence of the combined endpoint of all-cause mortality, stroke or myocardial infarction. In both the WARCEF and the COMMANDER HF study, anticoagulation did reduce the incidence of ischaemic stroke, suggesting that the lack of effect was not dose related. Our results confirm and extend upon the results of the COMMANDER HF trial and provide mechanistic underpinnings that explain the neutral outcomes. Furthermore, our findings suggest that clinically relevant doses of FXa inhibitors do not influence PAR mediated signalling and cardiac remodelling.

To the best of our knowledge, our study is the first to study the effect of Xa inhibition on cardiac remodelling in rats with established LV dysfunction. Several studies have, however, evaluated the early effect of FXa inhibition on myocardial ischaemia/reperfusion injury and post-infarct remodelling. The indirect FXa inhibitor fondaparinux has been shown to reduce infarct size following 2 h of reperfusion in a rat model of myocardial ischaemia-reperfusion [41]. The effects of FXa inhibitors are more variable as Flierl et al. did not observe infarct size reduction when rivaroxaban was given in rats with permanent coronary ligation [42]. However, a similar study by Bode et al. indicated that the administration of rivaroxaban immediately after surgery resulted in a reduction in infarct size and improvements in cardiac function. Conversely, when rivaroxaban was initiated 3 days after MI surgery, no effect on infarct size or cardiac function was observed [43]. Taken together, the available evidence on the effect of FXa inhibitors on cardiac function is in line with our observations and disputes their utility in chronic HF setting.

There are four PAR isoforms, but PAR1 and PAR2 are the predominant isoforms in the heart [13, 16]. PAR1 is activated by thrombin and FXa, but PAR2 is only activated by FXa. Both receptors are expressed in cardiomyocytes and cardiac fibroblast. Cleavage of PARs results in activation of several G protein–coupled receptors and their downstream signalling pathways, including RhoA/ROCK, the MAPK pathways, ERK 1/2 and ERK5 [16]. Several lines of evidence indicate that PARs are activated in HF and contribute to disease progression. First, PAR1 expression and the activity of its downstream signal transduction pathways are increased in murine models of chronic HF and in the ventricles of HF patients with ischaemic or idiopathic dilated cardiomyopathy [14, 15]. Second, the activation of PARs in cultured cardiomyocytes induces pathological hypertrophy, reflected by increases in cell size and sarcomeric organization, the activation of the foetal gene program and perturbations in cardiac calcium handling [28]. Third, PAR1 activation in cardiac fibroblasts induces a pro-fibrotic state reflected by enhanced proliferation and increased expression of transforming growth factor Beta (TGF-ß) [13, 28, 44]. Fourth, PAR1 activation is strongly pro-inflammatory as it induces the expression of interleukin (IL)-6, IL-8 and monocyte chemoattractant protein (MCP-1) [12, 13]. Yet, the most robust evidence comes from studies in PAR1-knockout (KO) mice and mice with overexpressing the PAR1. In a PAR1-KO mouse model, reduced cardiac remodelling was observed after I/R injury; however, infarct size was not affected. Accordingly, mice with cardiomyocyte-specific overexpression of PAR1 exhibited eccentric hypertrophy and dilated cardiomyopathy [16]. Additionally, PAR signalling activates RhoA/ROCK pathway [25, 45] which has been shown to mediate fibrosis in the heart [26]. Protein levels of ROCKs as well as RhoA activity were significantly increased in CHF patients [27]. Furthermore, the deletion of ROCK attenuates HF progression and improves the cardiac performance in mice [46, 47]. These findings provided a clear rationale for our studies.

However, in contrast to our expectations, we did not observe any changes in protein levels for PAR1 protein, nor did we detect differences in the activation of the AKT and ERK 1/2 signal transduction pathways downstream of PAR1 [48]. These findings are in consistence with a previous study [49] in which PAR1 protein expression did not differ at 12 weeks after MI induction or apixaban treatment. Moreover, it has been previously published that upregulated AKT exerts cardioprotective effects in models of preconditioning resulting in limiting infarct size [50, 51]. However, AKT is not regulated in a chronic ischaemia setting [52], and this finding was also seen in our study. Our observation that ERK1/2 protein levels were also not affected in post-MI LV dysfunction is in accordance with similar studies in post-MI LV dysfunction [53]. Consistent with other reports, we were able to detect a clear increase in RhoA activity after MI, but this was not affected by treatment with apixaban.

Other studies have demonstrated evidence that FXa inhibitors can influence PAR receptor signalling. For instance, Bukowska et al. showed that FXa inhibitor rivaroxaban reduced MAP kinase activity and diminished the upregulation of PARs, ICAM-1, LOX-1 and IL-8 and in human atrial tissue cultures in media containing activated FXa [54]. Interestingly, FXa has also been shown to activate ERK1/2 and induce pro-inflammatory cytokines in alveolar epithelial cells, which was suppressed by FXa inhibitor edoxaban [55]. In addition, the FXa inhibitor rivaroxaban did inhibit cardiac FXa activity and reduced cardiac fibrosis in a model of transverse aortic constriction [56]. Our results may therefore have been different in other disease models or if we would have induced a murine model prone to develop cardiac thrombi [57].

## Study Limitations

Despite strengths related to the direct measures of cardiac structure, function and other haematological parameters, our study does have limitations. First, in accordance with the previous study [49], we did not observe changes in PAR1 protein levels in post-MI HF model. Furthermore, the activity of downstream signalling pathways of the PAR1 was not increased after MI and apixaban did not alter this. It is possible that the activation of PAR1 and other thrombin-related pathways is more pronounced in other models or settings. Second, we started the treatment 2 weeks after MI when infarct healing had completed; we cannot exclude that FXa inhibition could be beneficial during earlier stages of post-infarct remodelling. Third, we employed apixaban at a dose that was within the safety range (FDA application no. 202155Orig1s000). Based on the AXA levels, this dosage is comparable with a 5-mg dose in humans [17]. The outcome of our study may have been different when a higher dose had been used. The clinical relevance of a study with high-dose apixaban is, however, limited as anticoagulants have a narrow therapeutic window and the benefits are often offset by the associated increased risk of bleeding events [39]. Our study does not exclude the possibility that thrombin-related pathways and PARs contribute to cardiac remodelling in HF. Our findings do, however, question the utility of FXa inhibitors as a pharmacological therapy to attenuate cardiac remodelling. Importantly, our study is in line with the neutral effects of the COMMANDER HF trial, and the molecular insights do not hint towards a direct of effect of apixaban on cardiac muscle structure and function.

## Conclusions

FXa inhibition with apixaban does not influence pathological cardiac remodelling after a MI. These data do not support the use of FXa inhibitor in HF patients with the aim to amend the severity of HF.

## Clinical Perspectives

It has been suggested that coagulation factors such as factor Xa (FXa) and thrombin promote maladaptive cardiac remodelling and could promote heart failure (HF) development via activation of the protease-activated receptors (PARs) in myocardial tissue. If this hypothesis is true, cardiac remodelling would be amendable by treatment with anticoagulants. To test this hypothesis, rats with HF after myocardial infarction (MI) were treated with the FXa inhibitor apixaban or a matching vehicle. While apixaban was effective in inhibiting FXa activity, it did not affect the activity of PAR1 signalling pathways, nor did it affect cardiac function and cardiac remodelling after MI. These findings are in line with the results of the recent COMMANDER HF trial and do not support the use of FXa inhibitors in HF patients with the aim to improve heart function or to modulate the clinical course of HF.

## Electronic Supplementary Material

ESM 1 (PDF 218 kb).

## References

[1] Zannad F (2018). Rising incidence of heart failure demands action. Lancet..

[2] Savarese G, Lund LH (2017). Global Public Health Burden of Heart Failure. Card Fail Rev.

[3] Ponikowski P, Voors AA, Anker SD, Bueno H, Cleland JGF, Coats AJS, et al. 2016 ESC Guidelines for the diagnosis and treatment of acute and chronic heart failure. Eur Heart J. 2016;37:2129–200.

[4] Lip GY, Gibbs CR (1999). Does heart failure confer a hypercoagulable state? Virchow’s triad revisited. J Am Coll Cardiol.

[5] Loh E, Sutton MSJ, Wun C-CC, Rouleau JL, Flaker GC, Gottlieb SS, Lamas GA, Moyé LA, Goldhaber SZ, Pfeffer MA (1997). Ventricular dysfunction and the risk of stroke after myocardial infarction. N Engl J Med.

[6] Abdul-Rahim AH, Perez A-C, Fulton RL, Jhund PS, Latini R, Tognoni G, Wikstrand J, Kjekshus J, Lip GY, Maggioni AP, Tavazzi L, Lees KR, McMurray J, Investigators of the Controlled Rosuvastatin Multinational Study in Heart Failure (CORONA), GISSI-Heart Failure (GISSI-HF) Committees and Investigators (2015). Risk of stroke in chronic heart failure patients without atrial fibrillation. Circulation..

[7] Kang S-H, Kim J, Park JJ, Oh I-Y, Yoon C-H, Kim H-J, Kim K, Choi DJ (2017). Risk of stroke in congestive heart failure with and without atrial fibrillation. Int J Cardiol.

[8] Adelborg K, Szépligeti S, Sundbøll J, Horváth-Puhó E, Henderson VW, Ording A, Pedersen L, Sørensen HT (2017). Risk of stroke in patients with heart failure. Stroke..

[9] Greenberg B, Neaton JD, Anker SD, Byra WM, Cleland JGF, Deng H, Fu M, la Police DA, Lam CSP, Mehra MR, Nessel CC, Spiro TE, van Veldhuisen DJ, vanden Boom CM, Zannad F (2019). Association of rivaroxaban with thromboembolic events in patients with heart failure, coronary disease, and sinus rhythm: a post hoc analysis of the COMMANDER HF trial. JAMA Cardiol.

[10] Zannad F, Greenberg B, Cleland JGF, Gheorghiade M, van Veldhuisen DJ, Mehra MR, Anker SD, Byra WM, Fu M, Mills RM (2015). Rationale and design of a randomized, double-blind, event-driven, multicentre study comparing the efficacy and safety of oral rivaroxaban with placebo for reducing the risk of death, myocardial infarction or stroke in subjects with heart failure and signi. Eur J Heart Fail.

[11] Westenbrink BD, Alings M, Connolly SJ, Eikelboom J, Ezekowitz MD, Oldgren J, Yang S, Pongue J, Yusuf S, Wallentin L, van Gilst WH (2015). Anemia predicts thromboembolic events, bleeding complications and mortality in patients with atrial fibrillation: insights from the RE-LY trial. J Thromb Haemost.

[12] Spronk HMH, de Jong AM, Crijns HJ, Schotten U, Van Gelder IC, ten Cate H (2014). Pleiotropic effects of factor Xa and thrombin: what to expect from novel anticoagulants. Cardiovasc Res.

[13] Spronk HMH, De Jong AM, Verheule S, De Boer HC, Maass AH, Lau DH (2017). Hypercoagulability causes atrial fibrosis and promotes atrial fibrillation. Eur Heart J.

[14] Moshal KS, Tyagi N, Henderson B, Ovechkin AV, Tyagi SC (2005). Protease-activated receptor and endothelial-myocyte uncoupling in chronic heart failure. Am J Physiol Heart Circ Physiol.

[15] Moshal KS, Tyagi N, Moss V, Henderson B, Steed M, Ovechkin A, Aru GM, Tyagi SC (2005). Early induction of matrix metalloproteinase-9 transduces signaling in human heart end stage failure. J Cell Mol Med.

[16] Pawlinski R, Tencati M, Hampton CR, Shishido T, Bullard TA, Casey LM, Andrade-Gordon P, Kotzsch M, Spring D, Luther T, Abe JI, Pohlman TH, Verrier ED, Blaxall BC, Mackman N (2007). Protease-activated receptor-1 contributes to cardiac remodeling and hypertrophy. Circulation..

[17] Shin H, Cho M-C, Kim RB, Kim C-H, Choi N-C, Kim S-K, Koh EH (2018). Laboratory measurement of apixaban using anti-factor Xa assays in acute ischemic stroke patients with non-valvular atrial fibrillation. J Thromb Thrombolysis.

[18] van der Meer P, Lipsic E, Henning RH, Boddeus K, van der Velden J, Voors AA, van Veldhuisen DJ, van Gilst WH, Schoemaker RG (2005). Erythropoietin induces neovascularization and improves cardiac function in rats with heart failure after myocardial infarction. J Am Coll Cardiol.

[19] Yurista SR, Silljé HHW, Oberdorf-Maass SU, Schouten E, Pavez Giani MG, Hillebrands J (2019). Sodium-glucose co-transporter 2 inhibition with empagliflozin improves cardiac function in non-diabetic rats with left ventricular dysfunction after myocardial infarction. Eur J Heart Fail.

[20] Takagawa J, Zhang Y, Wong ML, Sievers RE, Kapasi NK, Wang Y, Yeghiazarians Y, Lee RJ, Grossman W, Springer ML (2007). Myocardial infarct size measurement in the mouse chronic infarction model: comparison of area- and length-based approaches. J Appl Physiol.

[21] Osanai H, Ajioka M, Masutomi T, Kuwayama T, Ishihama S, Sakamato Y, Otaka N, Sakaguchi T, Inoue Y, Kanbara T, Nakashima Y, Asano H, Sakai K (2015). Measurement of anti-factor Xa activity in patients on apixaban for non-valvular atrial fibrillation. Circ J.

[22] Yurista SR, Silljé HHW, van Goor H, Hillebrands J-L, Heerspink HJL, de Menezes Montenegro L, et al. Effects of Sodium–Glucose Co-transporter 2 Inhibition with Empaglifozin on Renal Structure and Function in Non-diabetic Rats with Left Ventricular Dysfunction After Myocardial Infarction. Cardiovasc Drugs Ther. 2020;34:311–21. 10.1007/s10557-020-06954-6 PMC7242237 32185580

[23] Jin W, Goldfine AB, Boes T, Henry RR, Ciaraldi TP, Kim E-Y, et al. Increased SRF transcriptional activity in human and mouse skeletal muscle is a signature of insulin resistance. J Clin Invest. 2011;121:918–29. 10.1172/JCI41940 PMC3049368 21393865

[24] Van Blerk M, Bailleul E, Chatelain B, Demulder A, Devreese K, Douxfils J (2017). Influence of apixaban on commonly used coagulation assays: results from the Belgian national External Quality Assessment Scheme. Int J Lab Hematol.

[25] Greenberg DL, Mize GJ, Takayama TK. Protease-Activated Receptor Mediated RhoA Signaling and Cytoskeletal Reorganization in LNCaP Cells †. Biochemistry. 2003;42:702–9. 10.1021/bi027100x 12534282

[26] Lauriol J, Keith K, Jaffre F, Couvillon A, Saci A, Goonasekera SA, et al. RhoA signaling in cardiomyocytes protects against stress-induced heart failure but facilitates cardiac fibrosis. Sci Signal. 2014;7:ra100–ra100 10.1126/scisignal.2005262 PMC4300109 25336613

[27] Dong M, Liao JK, Fang F, Lee AP-W, Yan BP-Y, Liu M, Yu CM (2012). Increased Rho kinase activity in congestive heart failure. Eur J Heart Fail.

[28] Sabri A, Muske G, Zhang H, Pak E, Darrow A, Andrade-Gordon P, Steinberg SF (2000). Signaling properties and functions of two distinct cardiomyocyte protease-activated receptors. Circ Res.

[29] Gurbel PA, Tantry US (2014). Antiplatelet and anticoagulant agents in heart failure. JACC Heart Fail.

[30] Zeitler EP, Eapen ZJ (2015). Anticoagulation in heart failure: a review. J Atr Fibrillation.

[31] Gheorghiade M, Vaduganathan M, Fonarow GC, Greene SJ, Greenberg BH, Liu PP, Massie BM, Mehra MR, Metra M, Zannad F, Cleland JGF, van Veldhuisen DJ, Shah AN, Butler J (2013). Anticoagulation in heart failure: current status and future direction. Heart Fail Rev.

[32] Howell M (2001). Congestive heart failure and outpatient risk of venous thromboembolism: a retrospective, case-control study. J Clin Epidemiol.

[33] Weill-Engerer S, Meaume S, Lahlou A, Piette F, Saint-Jean O, Sachet A (2004). Risk factors for deep vein thrombosis in inpatients aged 65 and older: a case-control multicenter study. J Am Geriatr Soc.

[34] Melgaard L, Gorst-Rasmussen A, Lane DA, Rasmussen LH, Larsen TB, Lip GYH (2015). Assessment of the CHA 2 DS 2 -VASc score in predicting ischemic stroke, thromboembolism, and death in patients with heart failure with and without atrial fibrillation. JAMA..

[35] Mebazaa A, Spiro TE, Büller HR, Haskell L, Hu D, Hull R, Merli G, Schellong SW, Spyropoulos AC, Tapson VF, de Sanctis Y, Cohen AT (2014). Predicting the risk of venous thromboembolism in patients hospitalized with heart failure. Circulation..

[36] Jiao Z-Y, Zhang D-P, Xia K, Wang L-F, Yang X-C (2017). Clinical analysis of acute myocardial infarction caused by coronary embolism. J Thorac Dis.

[37] Raphael CE, Heit JA, Reeder GS, Bois MC, Maleszewski JJ, Tilbury RT, Holmes DR (2018). Coronary embolus. JACC Cardiovasc Interv.

[38] Gianstefani S, Douiri A, Delithanasis I, Rogers T, Sen A, Kalra S, Charangwa L, Reiken J, Monaghan M, MacCarthy P (2014). Incidence and predictors of early left ventricular thrombus after ST-elevation myocardial infarction in the contemporary era of primary percutaneous coronary intervention. Am J Cardiol.

[39] Homma S, Thompson JLP, Pullicino PM, Levin B, Freudenberger RS, Teerlink JR, Ammon SE, Graham S, Sacco RL, Mann DL, Mohr JP, Massie BM, Labovitz AJ, Anker SD, Lok DJ, Ponikowski P, Estol CJ, Lip GY, di Tullio MR, Sanford AR, Mejia V, Gabriel AP, del Valle M, Buchsbaum R, WARCEF Investigators (2012). Warfarin and aspirin in patients with heart failure and sinus rhythm. N Engl J Med.

[40] Zannad F, Anker SD, Byra WM, Cleland JGF, Fu M, Gheorghiade M, Lam CSP, Mehra MR, Neaton JD, Nessel CC, Spiro TE, van Veldhuisen D, Greenberg B, COMMANDER HF Investigators (2018). Rivaroxaban in patients with heart failure, sinus rhythm, and coronary disease. N Engl J Med.

[41] Macchi L, Ben MW, Guillou S, Tamareille S, Lamon D, Prunier D (2014). The synthetic pentasaccharide fondaparinux attenuates myocardial ischemia-reperfusion injury in rats via STAT-3. Shock..

[42] Flierl U, Fraccarollo D, Micka J, Bauersachs J, Schäfer A (2013). The direct factor Xa inhibitor Rivaroxaban reduces platelet activation in congestive heart failure. Pharmacol Res.

[43] Bode MF, Auriemma AC, Grover SP, Hisada Y, Rennie A, Bode WD, Vora R, Subramaniam S, Cooley B, Andrade-Gordon P, Antoniak S, Mackman N (2018). The factor Xa inhibitor rivaroxaban reduces cardiac dysfunction in a mouse model of myocardial infarction. Thromb Res.

[44] Sabri A, Short J, Guo J, Steinberg SF (2002). Protease-activated receptor-1-mediated DNA synthesis in cardiac fibroblast is via epidermal growth factor receptor transactivation: distinct PAR-1 signaling pathways in cardiac fibroblasts and cardiomyocytes. Circ Res.

[45] Klarenbach SW, Chipiuk A, Nelson RC, Hollenberg MD, Murray AG (2003). Differential actions of PAR2 and PAR1, in stimulating human endothelial cell exocytosis and permeability: the role of Rho-GTPases. Circ Res.

[46] Okamoto R, Li Y, Noma K, Hiroi Y, Liu PY, Taniguchi M, Ito M, Liao JK (2013). FHL2 prevents cardiac hypertrophy in mice with cardiac-specific deletion of ROCK2. FASEB J.

[47] Zhang YM, Bo J, Taffet GE, Chang J, Shi J, Reddy AK, Michael LH, Schneider MD, Entman ML, Schwartz RJ, Wei L (2006). Targeted deletion of ROCK1 protects the heart against pressure overload by inhibiting reactive fibrosis. FASEB J.

[48] Reséndiz JC, Kroll MH, Lassila R (2007). Protease-activated receptor-induced Akt activation--regulation and possible function. J Thromb Haemost.

[49] Shi G, Yang X, Pan M, Sun J, Ke H, Zhang C, Geng H (2018). Apixaban attenuates ischemia-induced myocardial fibrosis by inhibition of Gq/PKC signaling. Biochem Biophys Res Commun.

[50] HAUSENLOY D, YELLON D (2006). Survival kinases in ischemic preconditioning and postconditioning. Cardiovasc Res.

[51] Cantley LC (2002). The phosphoinositide 3-kinase pathway. Science.

[52] Ravingerová T, Matejíková J, Neckár J, Andelová E, Kolár F (2007). Differential role of PI3K/Akt pathway in the infarct size limitation and antiarrhythmic protection in the rat heart. Mol Cell Biochem.

[53] Tenhunen O, Soini Y, Ilves M, Rysä J, Tuukkanen J, Serpi R, Pennanen H, Ruskoaho H, Leskinen AH, Tenhunen O, Soini Y, Ilves M, Rysä J, Tuukkanen J, Serpi R, Pennanen H, Ruskoaho H, Leskinen AH (2006). p38 kinase rescues failing myocardium after myocardial infarction: evidence for angiogenic and anti-apoptotic mechanisms. FASEB J.

[54] Bukowska A, Zacharias I, Weinert S, Skopp K, Hartmann C, Huth C, Goette A (2013). Coagulation factor Xa induces an inflammatory signalling by activation of protease-activated receptors in human atrial tissue. Eur J Pharmacol.

[55] Bukowska A, Schild L, Bornfleth P, Peter D, Wiese-Rischke C, Gardemann A, Isermann B, Walles T, Goette A (2020). Activated clotting factor X mediates mitochondrial alterations and inflammatory responses via protease-activated receptor signaling in alveolar epithelial cells. Eur J Pharmacol.

[56] Guo X, Kolpakov MA, Hooshdaran B, Schappell W, Wang T, Eguchi S, Elliott KJ, Tilley DG, Rao AK, Andrade-Gordon P, Bunce M, Madhu C, Houser SR, Sabri A (2020). Cardiac expression of factor X mediates cardiac hypertrophy and fibrosis in pressure overload. JACC Basic Transl Sci.

[57] Bukowska A, Felgendreher M, Scholz B, Wolke C, Schulte JS, Fehrmann E, Wardelmann E, Seidl MD, Lendeckel U, Himmler K, Gardemann A, Goette A, Müller FU (2018). CREM-transgene mice: an animal model of atrial fibrillation and thrombogenesis. Thromb Res.

