# Supplementary material for: Factor Xa Inhibition with Apixaban Does Not Influence Cardiac Remodelling in Rats with Heart Failure After Myocardial Infarction
Source: Cardiovasc Drugs Ther. 2020 May 26;35(5):953–63. doi: 10.1007/s10557-020-06999-7 (PMC8452585; doi:10.1007/s10557-020-06999-7)
Supplement: Supplementary file 1 — (PDF 218 kb). [file 10557_2020_6999_MOESM1_ESM.pdf]

Supplemental Material

Factor Xa inhibition with apixaban does not influence cardiac remodeling in rats with heart failure after myocardial infarction

Salva R. Yurista MD, Herman H.W. Silljé PhD, Kirsten T. Nijholt BSc, Martin M. Dokter BASc, Dirk J. van Veldhuisen MD PhD, Rudolf A. de Boer MD PhD, B. Daan Westenbrink MD PhD

University Medical Center Groningen, University of Groningen, Department of Cardiology, Groningen, The Netherlands

Corresponding author:

B. Daan Westenbrink MD PhD

Department of Cardiology

University Medical Center Groningen

PO Box 30.001, Groningen 9700 RB,

The Netherlands

Phone : +31 50 361 2355 / Fax: +31 50 361 4391 / E-mail: b.d.westenbrink@umcg.nl

Supplementary Tables

Supplementary table S1. List of primers for qRT-PCR.

Genes	Forward primer sequence	Reverse primer sequence
**ANP**	ATGGGCTCCTTCTCCATCAC	TCTACCGGCATCTTCTCCTC
**BNP**	ACAATCCACGATGCAGAAGCT	GGGCCTTGGTCCTTTGAGA
**α-MHC**	GACAACTCCTCCCGCTTTGG	AAGATCACCCGGGACTTCTC
**β-MHC**	TCTGGAGGCCTTTGGCAATG	GATGCCAACTTTCCTGTTGC
**COL1A1**	ACAGCGTAGCCTACATGG	AAGTTCCGGTGTGACTCG
**TIMP1**	AGAGCCTCTGTGGATATGTC	CTCAGATTATGCCAGGGAAC
**36B4**	GTTGCCTCAGTGCCTCACTC	GCAGCCGCAAATGCAGATGG

ANP, atrial natriuretic peptide; BNP, brain natriuretic peptide; α-MHC, alpha-myosin heavy chain; β-MHC, alpha-myosin heavy chain; COL1A1, alpha-1 type I collagen; TIMP1, tissue inhibitor matrix metalloproteinase 1; 36B4, acidic ribosomal protein 36B4.

Supplementary table S2. List of primary antibodies for Western blot.

Antibody	Species/Clonality	Source (Catalog No.)
**phosphorylated-Akt**	Rabbit/Monoclonal	Cell signaling (#4060)
**Akt**	Rabbit/Monoclonal	Cell signaling (#4691)
**PAR1**	Mouse/Monoclonal	Santa Cruz (sc-13503)
**GAPDH**	Mouse/Monoclonal	Fitzgerald (10R-G109A)

ERK, Extracellular signal–regulated kinases; Akt, Protein Kinase B; PAR1, Protease-activated receptor; GAPDH, Glyceraldehyde 3-phosphate dehydrogenase

Supplementary Figure

Figure S1

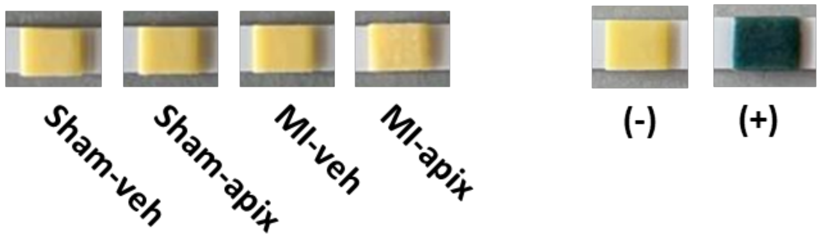

Figure S1. Urinary red blood cells measurements using dipstick analysis. Veh. vehicle; apix. apixaban; MI. myocardial infarction.
